# Continuous NU*track* monitoring revealed acute suppression and later changes in behavior after lipopolysaccharide challenge in nursery pigs

**DOI:** 10.1093/tas/txag122

**Published:** 2026-08-12

**Authors:** Aaron J Holliday, Lindsey E Hulbert, Benny E Mote, Majid Jaberi-Douraki, Brooke L Parrish, Storey L Forster, Larissa E Meier, Benjamin S Riggan, Lance C Pérez, Ty B Schmidt

**Affiliations:** Department of Animal Science, University of Nebraska, Lincoln, NE 68583, United States; Animal Science and Industry, Kansas State University, Manhattan, KS 66506, United States; Department of Animal Science, University of Nebraska, Lincoln, NE 68583, United States; Mathematics and Data Science, Kansas State University, Manhattan, KS 66506, United States; Department of Animal Science, University of Nebraska, Lincoln, NE 68583, United States; Department of Animal Science, University of Nebraska, Lincoln, NE 68583, United States; Department of Animal Science, University of Nebraska, Lincoln, NE 68583, United States; Electrical & Computer Engineering, University of Nebraska, Lincoln, NE 68583, United States; College of Engineering, University of Nebraska, Lincoln, NE 68583, United States; Department of Animal Science, University of Nebraska, Lincoln, NE 68583, United States

**Keywords:** nursery pigs, lipopolysaccharide, sickness behavior, NU*track*, precision livestock technology, continuous behavior monitoring

## Abstract

This study evaluated changes in the active and resting behaviors of group-housed, newly weaned pigs challenged with lipopolysaccharide (LPS). At weaning (∼21 days of age), pigs (*n* = 192; 5.73 ± 1.8 kg) were stratified by sex, litter, and body weight and randomly assigned to 1 of 3 treatments (16 pigs/pen; 4 pens/treatment): (i) saline-injected control (SAL); (ii) 50%-LPS, pens in which 8 of 16 pigs received LPS and 8 of 16 pigs received saline; or (iii) 100%-LPS, pens in which all pigs received LPS. Following a 9-d acclimation period, assigned injections were administered on study day 10. Pigs in the SAL treatment group and 32 pigs in the 50%-LPS treatment group received a 3.0-mL subcutaneous saline injection. The remaining pigs in the 50%-LPS treatment group and all pigs in the 100%-LPS treatment group received a 3.0-mL subcutaneous LPS injection (*Escherichia* coli O111: B4; 300 µg/kg BW). Behavior was continuously monitored for 42 d using the NUtrack system to quantify meters walked, body revolutions, time spent standing, time at the feeder, time lying laterally and sternal, total time lying, and time sitting. During the LPS response period (days 10 to 13), pigs in the 100%-LPS and 50%-LPS treatment groups displayed a clear sickness-behavior response characterized by reduced activity and increased resting behavior (*P* ≤ 0.05). Compared with SAL, pigs in the LPS treatment groups walked fewer meters, completed fewer revolutions, spent less time standing and at the feeder, and spent more time lying and resting (*P* ≤ 0.05). At the end of the recovery period (day 19) and during the early post-recovery period, pigs in both LPS treatment groups walked more, completed more revolutions, and spent more time standing and at the feeder than pigs in the SAL treatment group (*P* ≤ 0.05). These later differences occurred primarily on study days 19 to 25, corresponding to 9 to 15 d after challenge. Despite these later increases in activity, pigs in the 100%-LPS treatment group had reduced nursery exit BW, reduced ADG, and less total weight gain than pigs in the SAL treatment group (*P* ≤ 0.05). These findings indicate that the behavioral response to LPS challenge extended beyond the LPS response period and included later changes in activity that were detected through continuous behavioral monitoring.

## Teaser

Continuous NU*track* monitoring showed that an LPS challenge in nursery pigs caused clear short-term reductions in activity and increases in resting behaviors, followed by later increases in activity that emerged well after the acute sickness period. These findings demonstrate that continuous behavioral monitoring can detect both immediate and delayed responses to immune challenge in group housed pigs.

## Introduction

Timely and accurate identification of sick pigs is critical for minimizing the spread of pathogens, reducing morbidity and mortality, and ensuring high standards of welfare. Rapid and accurate intervention can improve treatment outcomes, support optimal growth, and ultimately enhance overall production efficiency (Matthews et al. 2016). However, detection of illness still relies mainly on human observation, which depends on the presence of visible clinical signs such as depression, lethargy, isolation, reduced feed intake, diarrhea, or labored breathing. These symptoms typically only become apparent after a pathogen has spread beyond the initial site of infection and triggered a systemic proinflammatory response (López and Hermesh 2011). By this stage, inflammation is well advanced, potentially reducing the effectiveness of interventions.

Early disease detection is further hindered by the evolved behavior of pigs. As a prey species, pigs may suppress outward signs of illness or weakness when exposed to perceived threats, including the presence of human caretakers. This natural tendency makes early detection through visual inspection particularly difficult, as pigs may appear alert and active while experiencing physiological stress or inflammation (Underwood 2002; Weary et al. 2009). Furthermore, subtle changes in posture, activity, or behavior may be difficult, if not impossible, to recognize through casual human observation (Bortoluzzi et al. 2023).

The scale of modern U.S. pork production systems adds another layer of complexity, as large farrow to finish systems require only ∼2.2 full time caretakers per 10,000 hogs raised, meaning each employee may be responsible for overseeing several thousand pigs (NPPC 2024). If visual assessment of the pigs were the sole responsibility, a caretaker would have approximately five seconds per pig over an eight-hour workday. In reality, caretakers’ daily job requirements not only include visually monitoring the pigs but also managing the facility, which involves tasks such as feeding, cleaning, and maintenance, further reducing the time available for health evaluations. Compounding the problem, the clinical symptoms used for health decisions, such as scours, coughing, or gaunt appearance, often reflect disease progression rather than onset (Gemus-Benjamin and Kramer 2014). This places the burden on caretakers to detect early, non-specific indicators of illness in pigs that are biologically predisposed to mask signs of illness. Even with training and experience, visual assessments under these conditions can remain subjective and vary among observers, creating the potential for inconsistent detection of early disease-related changes (Czycholl et al. 2016; Matthews et al. 2016). This disconnect between physiological condition, and observable behavior represents a significant limitation to the timely and effective implementation of interventions.

In pigs, illness is often accompanied by behavioral changes such as reduced locomotion, prolonged lying, diminished interest in feed and water, and decreased interaction with pen mates. These shifts can emerge within hours of immune activation and often precede the appearance of visible clinical symptoms (Touchette et al. 2002; Williams et al. 2009). While researchers have employed scan sampling and video analysis to document such behaviors under controlled conditions (Escobar et al. 2007; Veit et al. 2020), these techniques are time-intensive and have limited scope. Furthermore, to achieve high precision and accuracy, a well-trained technician in either veterinary medicine or applied ethology is required (Bortoluzzi et al. 2023). Regardless of the setting or environment, capturing early and transient behavioral changes requires a level of continuous monitoring that cannot be achieved solely through manual observation. Subtle shifts in activity may last only minutes or occur sporadically, making these changes easy to miss even with frequent visual checks. As a result, early indicators of illness are often underreported or entirely overlooked, particularly without the use of automated tracking systems.

This limitation has led to increased interest in precision livestock technology as a tool to improve early disease detection. For precision livestock technology systems to be effective in identifying pigs undergoing immune challenge or injury, three key criteria must be met: (i) continuous and individual identification of animals, (ii) reliable tracking of behavioral metrics over time, and (iii) detection of meaningful deviations from expected patterns (Berckmans 2006, 2017). One system designed to meet these criteria is the NU*track* Livestock Monitoring system, a computer vision platform that uses 4K resolution security cameras and machine learning algorithms to continuously monitor the activity and posture of individual pigs within group housing environments. NU*track* enables the quantification of both active behaviors (meters walked/d, body revolutions, time spent standing, and time spent at the feeder) and resting behaviors (lying in a lateral or sternal position and sitting), providing an objective and scalable means of detecting early behavioral changes associated with immune activation or physiological stress (Psota et al. 2019, 2020a, 2020b, 2023a, 2023b; Schmidt et al. 2022).

The long-term goal of this research is to determine whether behavioral patterns can be used to identify compromised pigs. Toward this goal, the objective of the present study was to evaluate changes in the active and resting behaviors of nursery pigs during the acute immune response to an endotoxin challenge and throughout the subsequent recovery and post-recovery periods. To address this objective, a lipopolysaccharide (LPS) model was used to induce transient immune activation, and behavior was continuously monitored with the NU*trac*k system throughout a 42-d nursery period.

## Materials and methods

### Animal and experimental design

All experimental procedures complied with the *Guide for the Care and Use of Agricultural Animals in Research and Teaching* and were approved by the Institutional Animal Care and Use Committee at the University of Nebraska (IACUC #1832).

**Figure 1:**
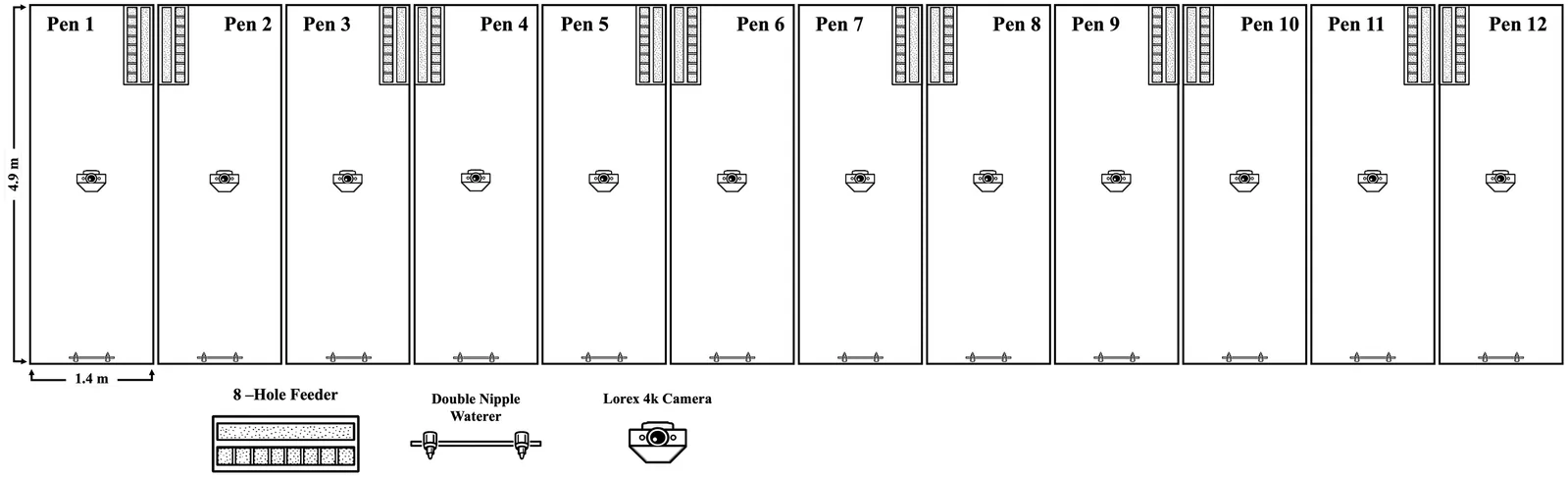
Schematic of the 12 nursery pens and placement of NU*track* system within the University of Nebraska—Lincoln Eastern Nebraska Research, Extension, and Education Center Swine unit, Mead, NE.

Using 192 pigs weaned at ∼21 d of age (5.73 ± 1.8 kg), this study was designed to evaluate the effects of a lipopolysaccharide challenge on the behavior of group-housed nursery pigs. Pigs were selected from 20 litters at the University of Nebraska—Lincoln Eastern Nebraska Research, Extension, and Education Center Swine unit. For the study, pigs were housed in a single room with 12 pens. Each pen (4.9 × 1.4 m) was equipped with an 8-hole feeder and traditional nipple waterers, providing pigs with ad libitum access to feed (formulated to meet or exceed Nutrient Requirements of Swine, Table 1 [NRC 2012]) and water. One Nocturnal 3 security camera (4K Ultra High Definition, featuring color night vision, infrared night vision, and power-over-Ethernet; Lorex Technology Inc., Markham, Canada) was positioned facing downward from the ceiling in the center of each pen (Fig. 1).

**Table 1:** Diet composition fed to nursery pigs assigned to 100%-LPS, all pigs in the pen received 300 µg/kg BW lipopolysaccharide; 50%-LPS, pens contained a 1:1 ratio of LPS challenged to saline injected pigs; or SAL, all pigs in the pen received saline, during the nursery period.

Ingredient (% of diet)^a^	Starter	Nursery 1	Nursery 2^b^	Nursery 3
**Corn**	43.0	43.6	60.0	57.0
**Soybean Meal**	14.7	32.0	33.8	34.8
**Dried Whey**	22.5	15.0		
**Tallow**				
**Fish Meal**	8.00	4.0		
**Animal Plasma**	6.00			
**Corn Oil**	3.00	3.0	3.00	3.0
**Dicalcium Phosphate**	0.40	1.0	1.70	1.7
**Limestone**	0.25	0.35	0.60	0.30
**Salt**	0.30	0.30	0.30	0.30
**Vitamin Premix** ^c^	0.25	0.25	0.25	0.15
**Swine TM Premix** ^d^	0.15	0.15	0.15	0.15
**Mecadox**	1.0			
**Zinc Oxide**	0.4	0.3		
**DL-Methionine**	0.05	0.025	0.025	0.025
**L-lysine HCL**			0.040	0.040
**Denegard**				0.180
**Aureomycin 50**				0.400
**Phytase**				
**Composition, %**				
**Net Energy**	2,631	2,534	2,515.0	2,531.0
**Crude Protein**	16.69	20.02	17.92	17.95
**Lysine**	1.33	1.21	1.03	1.03

a All ingredients reported on as a percentage as fed basis.

b Medicated Nursery 2 diet included Denegard and Auromycin 50.

c Vitamin Premix ingredients: Vitamin A, Vitamin D, Vitamin E, Vitamin K, Niacin, Panothenic Acid, Riboflavin, Vitamin B12.

d Swine TM Premix ingredients: Copper, Iodine, Iron, Manganese, Selenium, Zinc.

**Figure 2:**
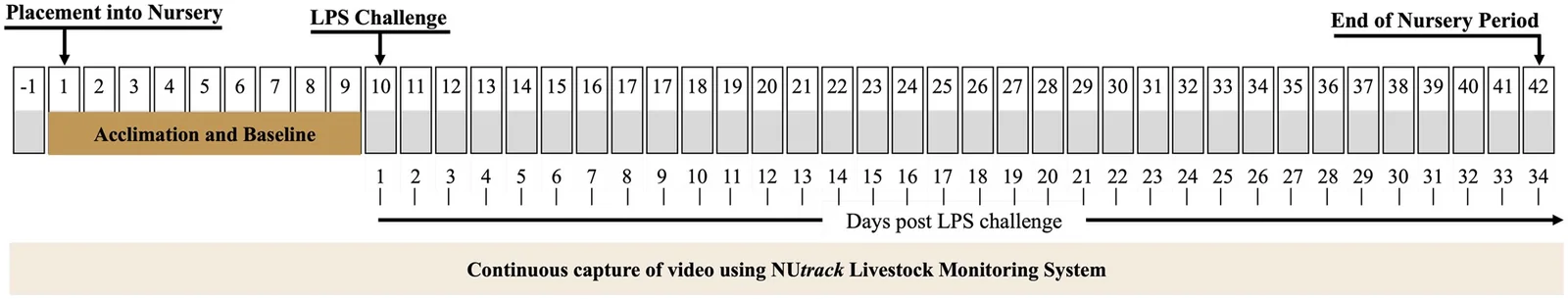
Timeline for the nursery period and the LPS Challenge.

At weaning, pigs were weighed, provided a color-coded/alpha-numerical ear tag (Hog Max tags from Allflex [Rahway, NJ, USA]), stratified by sex, litter, and weaning weight, and then randomly assigned to one of three treatments: (i) SAL—all pigs in the pen received a subcutaneous saline injection; (ii) 50%-LPS—half of the pigs in each pen (8 of 16) received an LPS injection and the other half received subcutaneous saline injection; or (iii) 100%-LPS—all pigs in the pen received an LPS injection. Thus, the 50%-LPS treatment represented a mixed pen environment in which challenged and non-challenged pigs remained housed together within the same pen. Following placement into pens (16 pigs/pen, 4 pens/treatment), pigs were continuously monitored using the NU*track* system for the duration of the study (42 days), including a nine-day acclimation period. On day 10, pigs were weighed at 0800 h, and the assigned treatments were administered (timeline for the nursery period and the LPS challenge is shown in Fig. 2). All pigs in the SAL treatment and 32 in the 50% treatment (randomly selected, 8 pigs/pen) received a 3.0 mL/pig subcutaneous sham injection (saline, no LPS). The remaining pigs in the 50%-LPS treatment (8 pigs/pen) and all in the 100%-LPS treatment received a 3.0 mL/pig subcutaneous LPS (*Escherichia coli* O111: B4; Sigma Aldrich, St. Louis, MO, USA) dissolved in sterile saline calculated to challenge each pig with 300 µg/kg of body weight collected on day 10. All injections were administered subcutaneously in the left medial inguinal area using a syringe with a 1.27 cm long 21-gauge needle. Immediately following the administration of the LPS challenge, the research team maintained continuous onsite monitoring for 4 hours using live observation by a trained veterinarian and ethologist (Bortoluzzi et al. 2023). Any LPS-challenged pig that became unresponsive to external stimuli was administered a rescue intervention protocol, prepared by the University of Nebraska–Lincoln IACUC attending veterinarian, consisting of 0.5 mL/pig epinephrine and 0.5 mL/pig dantrolene intramuscularly. Nine pigs required rescue intervention, including five pigs from the 100%-LPS treatment group and four pigs from the 50%-LPS treatment group. None of the nine pigs responded to treatment, and all were humanely euthanized in accordance with IACUC guidelines. Observations collected after removal were treated as missing in the repeated-measures analyses.

Behavioral data were collected using the NU*track* system, a computer vision platform developed at the University of Nebraska–Lincoln for continuous tracking of individual pigs housed in groups (Psota et al. 2019). NU*track* is a deep learning–based, multi-object tracking system that achieves greater than 95% precision and recall in maintaining long-term identification and location of individual pigs. The system consists of overhead Nocturnal-3 security camera, placed at a 90° over each pen, a Dell-Alienware processing unit (Round Rock, TX, USA) equipped with an NVIDIA graphics processor (Santa Clara, CA, USA), and proprietary software developed specifically for livestock behavior tracking (Psota et al. 2019, 2020a, 2020b, 2023a, 2023b). The video was recorded using a 4K capable camera at five frames per second and 4 MP resolution (2688 x 1520). The application of NU*track* for evaluating behavior in group-housed pigs was reported by Schmidt et al. (2022), Bortoluzzi et al. (2023) and Obermier et al. (2023).

Details regarding the NU*track* system’s software are reported in Psota et al. (2019, 2020a, 2020b, 2023a, 2023b). In brief, this method for long-term tracking of individual pigs within a group-housed setting is feasible via deep convolutional neural networks. These networks locate individual targets (pigs) within a fixed living space and classify identity (Psota et al. 2019). This detection utilizes deep learning from human-annotated images, in which it joins body parts (left ear, right ear, shoulder, and tail) into “instances” via part association vectors (Psota et al. 2019). Additionally, activity tracking is processed via the Bayesian multi-object tracking method. Using frame-to-frame movement probabilities with images captured at five frames per second, probabilities of individual identification (by the NU*track* ear tags) can be assessed. Data captured across various environments indicates that this method is over 90% accurate in maintaining individual identification (Psota et al. 2019). NU*track* does not directly classify discrete pig behaviors. Instead, the system quantifies activity and body posture from the detected positions of individual body parts and changes in location across successive video frames.

**Figure 3:**
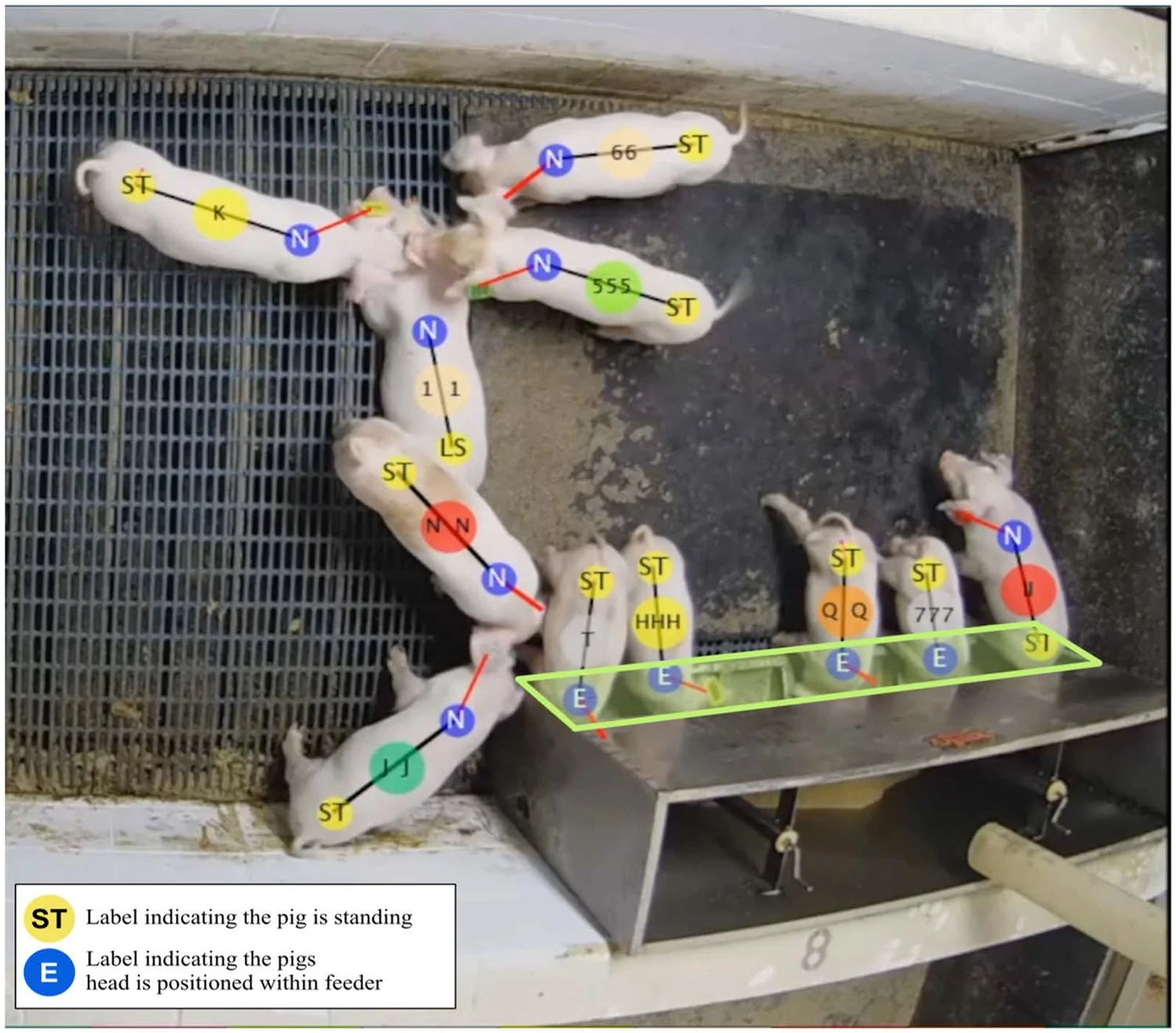
The NU*track* system classified the behavior “*at the feeder*” by the accumulated amount of time an individual pig was classified as standing (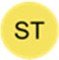) with the head positioned appropriately for eating (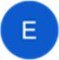) within a designated aspect of the feeder (transparent green rectangle identified below).

The NU*track* system has been reported to be successful at continuously tracking various social and normal behaviors (Schmidt et al. 2022). For this study, the determined activities include revolutions (radians), distance traveled (meters), time spent at the feeder (seconds), time spent lying lateral (seconds), time spent lying sternal (seconds), time spent sitting (seconds), and time spent standing (seconds), which were all trained on human-annotated images and videos. Behavioral outputs were validated by comparing NU*track* data to manually scored videos by trained human observers, confirming a high degree of agreement and allowing the recorded behaviors to be considered representative of actual pig activity. Revolutions quantify the extent of rotational body movement per day, calculated by summing the angular changes in body orientation (in radians) and converting the total to revolutions using the formula: revolutions = radians × 0.159155. Times associated with activities of standing, at the feeder (Fig. 3), lying lateral, lying sternal, and total time lying were converted to time/day associated with each activity. Active behaviors included daily distance traveled, revolutions, daily duration of time standing, and at the feeder. Resting behaviors included daily durations of lying lateral, lying sternal, and sitting.

### Statistical analysis

Active and resting behaviors were analyzed using the PROC GLIMMIX procedure of SAS (SAS Inst. Inc., Cary, NC) by trial day and post-challenge period. Study days were reported relative to weaning, with day 1 representing the day of weaning and the LPS challenge administered on day 10. For the analysis by trial day, fixed effects included treatment, day, sex, and the treatment × day interaction. Pen was included as a random effect and served as the experimental unit for the treatment comparison, and individual pig served as the observational unit and repeated-measures subject. Because treatment was assigned at the pen level, denominator degrees of freedom for treatment comparisons were based on pen. Day was treated as a repeated measure within pig using a first-order autoregressive (AR[1]) covariance structure for meters walked, body revolutions, time at the feeder, time lying lateral, time lying sternal, and time sitting. A compound-symmetry covariance structure was used for time spent standing and total time lying because the AR(1) models did not converge for these outcomes.

The pre-challenge baseline period consisted of days 1 to 9 and was evaluated using the trial-day analysis. Post-challenge periods were identified based on distinct, biologically relevant patterns observed in the active-behavior data. These periods included the LPS response period from days 10 to 13, the recovery period from days 14 to 19, the early post-recovery period from days 20 to 25, and the late post-recovery period from days 26 to 42. For the period analyses, observations within each period were averaged for each pig to generate one mean value per behavior per period. Fixed effects included treatment, period, and the treatment × period interaction, with pen included as a random effect, individual pig as the repeated-measures subject, and a compound-symmetry covariance structure.

For both the trial day and period analysis, denominator degrees of freedom were adjusted using the Kenward–Roger method. Observations collected after removal of the nine pigs described above were treated as missing in the repeated-measures analyses. When treatment interactions were significant (*P* < 0.05), treatment LSMeans were compared within day or period using the SLICE and SLICEDIFF options of SAS with Tukey adjustment. Data are presented as LSMeans ± SEM. Three-way interactions involving sex and litter were not significant (*P* > 0.18) and were excluded from the results.

## Results

### Meters walked

**Figure 4:**
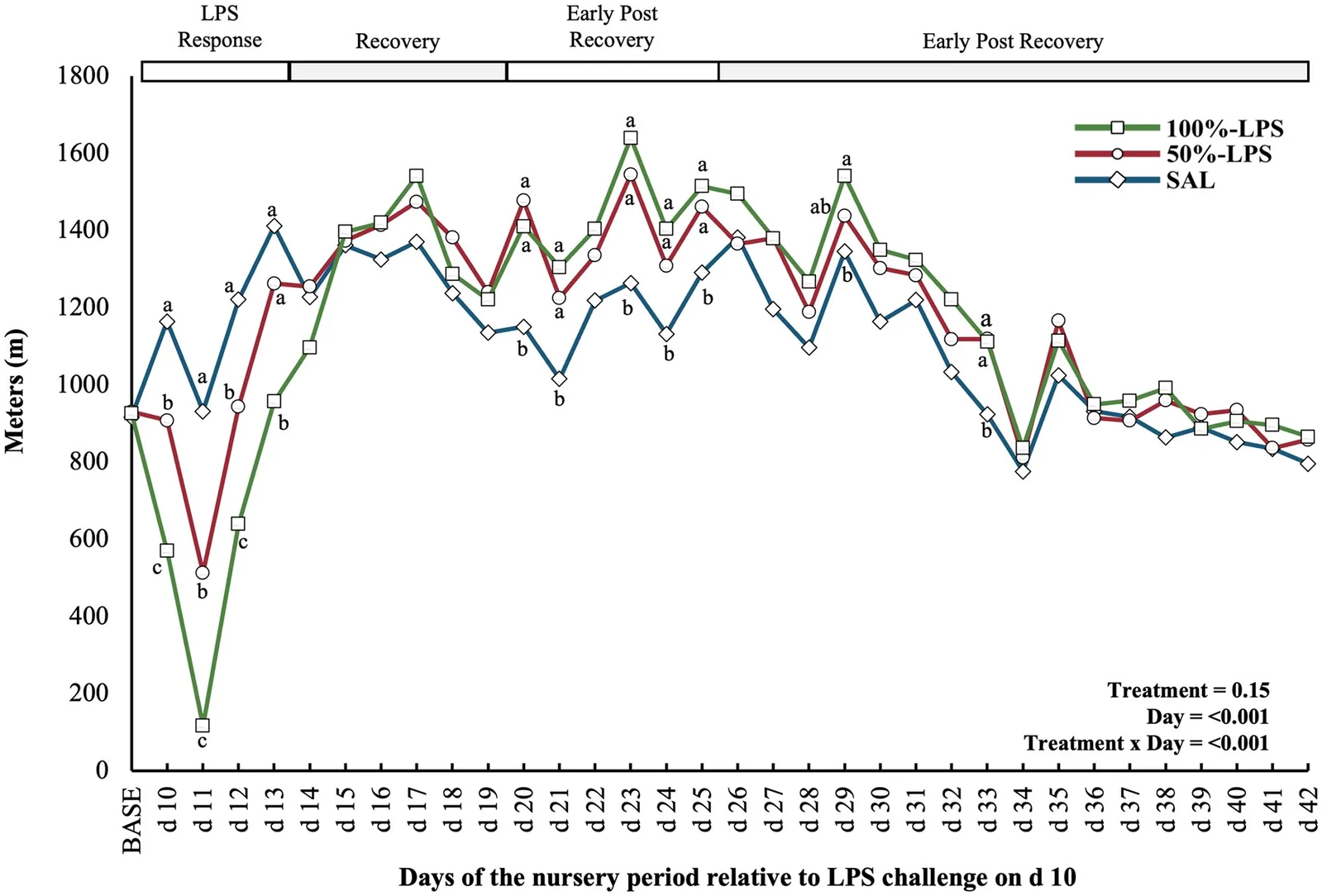
Meters walked/day by nursery pigs assigned to 100%-LPS, all pigs in the pen received 300 µg/kg BW lipopolysaccharide; 50%-LPS, pens contained a 1:1 ratio of LPS challenged to saline injected pigs; or SAL, all pigs in the pen received saline, across the 42-d nursery period.

There was an effect of day (*P* < 0.001) and a treatment × day interaction (*P* < 0.001) for meters walked/day; however, there was no treatment effect (*P* = 0.15). When evaluated across the four post-challenge periods, there was an effect of period (*P* < 0.001) and a treatment × period interaction (*P* < 0.001), but no treatment effect (*P* = 0.13). Meters walked/day did not differ among the three treatments prior to the LPS challenge (*P* > 0.99). During the LPS response (days 10 to 13), pigs in the 100%-LPS, 50%-LPS, and SAL treatment groups walked 567, 886, and 1,172 m/day, respectively (SEM = 27.6; Fig. 4). Pigs in the 100%-LPS treatment group walked fewer (*P* < 0.001) meters/day than pigs in the 50%-LPS and SAL treatment groups, and pigs in the 50%-LPS group walked fewer (*P* < 0.001) meters/day than pigs in the SAL treatment group. These treatment differences occurred on days 10 to 12; on day 13, pigs in the 100%-LPS treatment group continued to walk fewer (*P* < 0.001) meters than pigs in the 50%-LPS and SAL treatment groups, whereas the 50%-LPS and SAL treatment groups did not differ (*P* = 0.87). During the recovery period (days 14 to 19), there was no treatment difference (*P* = 0.10) for the period mean, and no differences were observed among treatment groups on individual days (*P* ≥ 0.56).

During the early post-recovery period (days 20 to 25), pigs in both the 100%-LPS and 50%-LPS treatment groups walked more (*P* < 0.001) meters/day than pigs in the SAL treatment group, with no difference (*P* = 0.29) between the two LPS treatment groups. Within this period, pigs in both LPS treatment groups walked more (*P* < 0.001) meters/day than pigs in the SAL treatment group on days 20 and 21, whereas there were no differences (*P* ≥ 0.11) among treatment groups on day 22. On days 23 to 25, pigs in both LPS treatment groups again walked more (*P* < 0.001) meters/day than pigs in the SAL treatment group. During the late post-recovery period (days 26 to 42), pigs in the 100%-LPS treatment group walked more (*P* = 0.03) meters/day than pigs in the SAL treatment group, whereas pigs in the 50%-LPS treatment group were intermediate. Within this period, treatment differences were limited to days 29 and 33, with no differences (*P* ≥ 0.84) observed among treatment groups from days 34 to 42 (Fig. 4).

### Body revolutions

**Figure 5:**
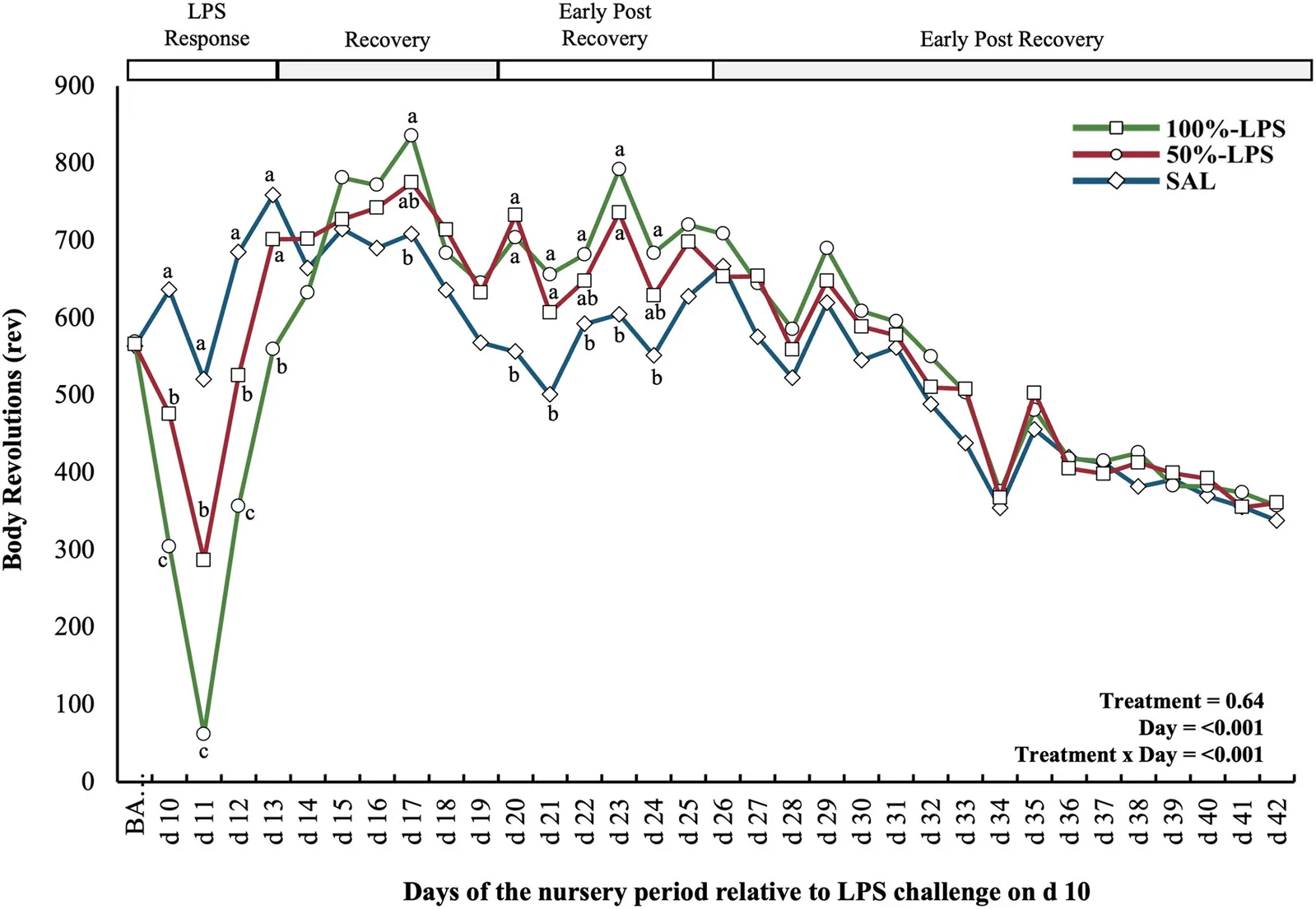
Body revolutions/day by nursery pigs assigned to 100%-LPS, all pigs in the pen received 300 µg/kg BW lipopolysaccharide; 50%-LPS, pens contained a 1:1 ratio of LPS challenged to saline injected pigs; or SAL, all pigs in the pen received saline, across the 42-d nursery period.

There was an effect of day (*P* < 0.001) and a treatment × day interaction (*P* < 0.001) for body revolutions/day; however, there was no treatment effect (*P* = 0.64). When evaluated across the four post-challenge periods, there was an effect of period (*P* < 0.001) and a treatment × period interaction (*P* < 0.001), but no treatment effect (*P* = 0.16). During the baseline period (days 1 to 9), there were no differences (*P* = 0.99) among the three treatment groups, which averaged 566.3 revolutions/pig/day (Fig. 5). During the LPS response (days 10 to 13), pigs in the 100%-LPS, 50%-LPS, and SAL treatment groups completed 320, 489, and 650 revolutions/day, respectively (SEM = 13.4). Pigs in the 100%-LPS treatment group completed fewer (*P* < 0.001) revolutions than pigs in the 50%-LPS and SAL treatment groups, and pigs in the 50%-LPS treatment group completed fewer (*P* < 0.001) revolutions than pigs in the SAL treatment group. Within this period, all three treatment groups differed (*P* < 0.001) on days 10 to 12. On day 13, pigs in the 100%-LPS treatment group completed fewer (*P* < 0.001) revolutions than pigs in the 50%-LPS and SAL treatment groups, whereas the 50%-LPS and SAL treatment groups did not differ (*P* = 0.65). During the recovery period (days 14 to 19), pigs in the 100%-LPS, 50%-LPS, and SAL treatment groups completed 728, 715, and 664 revolutions/day, respectively (SEM = 13.7). Pigs in the 100%-LPS and 50%-LPS treatment groups completed more (*P* ≤ 0.02) revolutions/day than pigs in the SAL treatment group, whereas the two LPS treatment groups did not differ (*P* = 0.78). Within this period, treatment differences were limited to day 17, when pigs in the 100%-LPS treatment group completed more (*P* < 0.001) revolutions than pigs in the SAL treatment group, whereas pigs in the 50%-LPS treatment group were intermediate. There were no differences (*P* ≥ 0.25) among treatment groups on days 18 and 19.

During the early post-recovery period (days 20 to 25), pigs in the 100%-LPS, 50%-LPS, and SAL treatment groups completed 709, 674, and 565 revolutions/day, respectively (SEM = 13.8). Pigs in both LPS treatment groups completed more (*P* < 0.001) revolutions/day than pigs in the SAL treatment group, whereas the two LPS treatment groups did not differ (*P* = 0.17). Within this period, pigs in both LPS treatment groups completed more (*P* ≤ 0.002) revolutions than pigs in the SAL treatment group on days 20, 21, and 23. On days 22 and 24, pigs in the 100%-LPS treatment group completed more (*P* ≤ 0.05) revolutions than pigs in the SAL treatment group, whereas pigs in the 50%-LPS treatment group were intermediate. There was no difference (*P* = 0.35) among treatment groups on day 25. During the late post-recovery period (days 26 to 42), there was a tendency for a treatment difference (*P* = 0.06) in revolutions/day, although no differences (*P* ≥ 0.35) were observed among treatment groups on individual days (Fig. 5).

### Time standing

**Figure 6:**
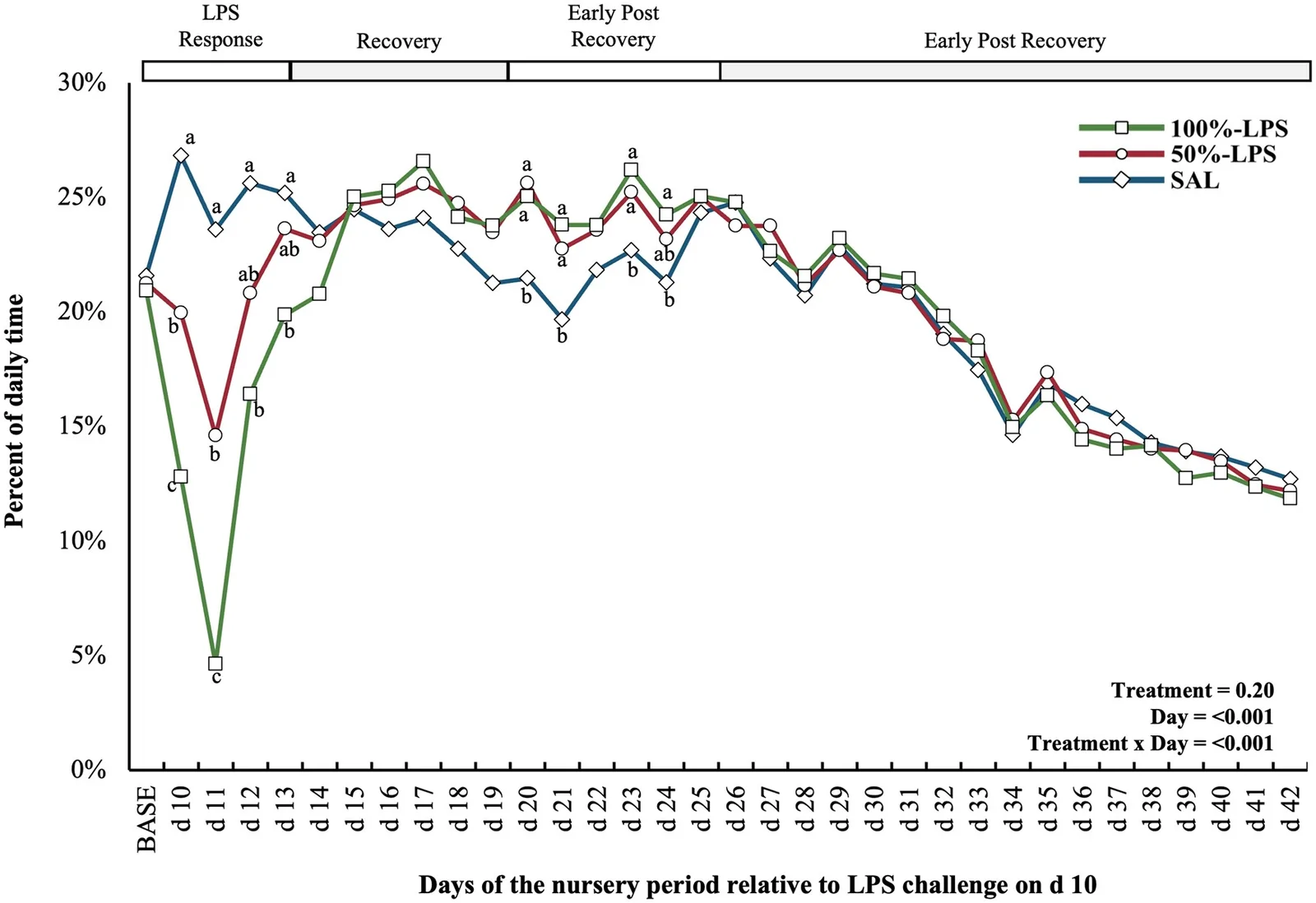
Time spent standing/day by nursery pigs assigned to 100%-LPS, all pigs in the pen received 300 µg/kg BW lipopolysaccharide; 50%-LPS, pens contained a 1:1 ratio of LPS challenged to saline injected pigs; or SAL, all pigs in the pen received saline, across the 42-d nursery period.

There was an effect of day (*P* < 0.001) and a treatment × day interaction (*P* < 0.001) for the percentage of daily time spent standing; however, there was no treatment effect (*P* = 0.20). When evaluated across the four post-challenge periods, there were effects of treatment (*P* < 0.001), period (*P* < 0.001), and a treatment × period interaction (*P* < 0.001). During the baseline period (days 1 to 9), there were no differences (*P* = 0.98) among the three treatment groups in the percentage of daily time spent standing. During the LPS response (days 10 to 13), pigs in the 100%-LPS, 50%-LPS, and SAL treatment groups spent 13.3, 19.3, and 25.1% of daily time standing, respectively (SEM = 0.44; Fig. 6). Pigs in the 100%-LPS treatment group spent less (*P* < 0.001) time standing than pigs in the 50%-LPS and SAL treatment groups, and pigs in the 50%-LPS treatment group spent less (*P* < 0.001) time standing than pigs in the SAL treatment group. Within this period, all three treatment groups differed (*P* < 0.001) on days 10 and 11. On days 12 and 13, pigs in the 100%-LPS treatment group spent less (*P* < 0.001) time standing than pigs in the 50%-LPS and SAL treatment groups. There was no treatment difference during the recovery period (*P* = 0.06), and no differences (*P* ≥ 0.33) were observed among treatment groups on individual days 14 to 19.

During the early post-recovery period (days 20 to 25), pigs in the 100%-LPS, 50%-LPS, and SAL treatment groups spent 24.8, 24.2, and 21.5% of daily time standing, respectively (SEM = 0.45). Pigs in both the 100%-LPS and 50%-LPS treatment groups spent more (*P* < 0.001) time standing than pigs in the SAL treatment group, whereas the 100%-LPS and 50%-LPS treatment groups did not differ (*P* = 0.62). Within this period, pigs in 100%-LPS and 50%-LPS treatment groups spent more (*P* < 0.001) time standing than pigs in the SAL treatment group on days 20, 21, and 23. There were no differences (*P* ≥ 0.11) among the three treatment groups on day 22. On day 24, pigs in the 100%-LPS treatment group spent more (*P* < 0.001) time standing than pigs in the SAL treatment group, whereas pigs in the 50%-LPS treatment group were intermediate. There was no difference (*P* ≥ 0.26) among treatment groups on day 25. During the late post-recovery period (days 26 to 42), there was no treatment difference (*P* = 0.89), and no differences (*P* ≥ 0.26) were observed among treatment groups on individual days (Fig. 6).

### Time at the feeder

**Figure 7:**
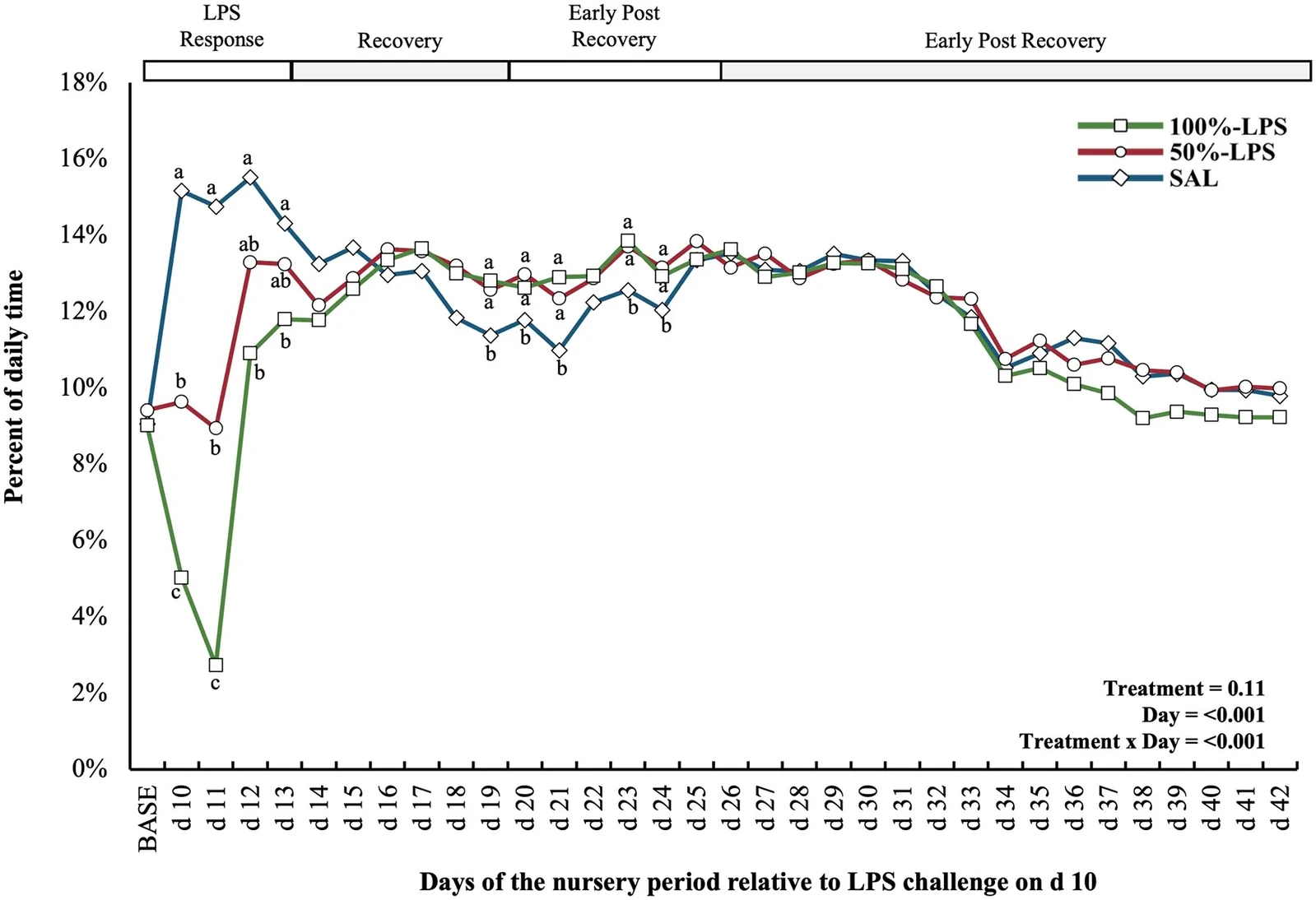
Time spent at the feeder/day by nursery pigs assigned to 100%-LPS, all pigs in the pen received 300 µg/kg BW lipopolysaccharide; 50%-LPS, pens contained a 1:1 ratio of LPS challenged to saline injected pigs; or SAL, all pigs in the pen received saline, across the 42-d nursery period.

There was an effect of day (*P* < 0.001) and a treatment × day interaction (*P* < 0.001) for the percentage of daily time spent at the feeder; however, there was no treatment effect (*P* = 0.11). When evaluated across the four post-challenge periods, there were effects of treatment (*P* < 0.001), period (*P* < 0.001), and a treatment × period interaction (*P* < 0.001). During the baseline period (days 1 to 9), there were no differences (*P* ≥ 0.93) among treatment groups in time spent at the feeder. During the LPS response (days 10 to 13), pigs in the 100%-LPS, 50%-LPS, and SAL treatment groups spent 7.4, 11.0, and 14.8% of daily time at the feeder, respectively (SEM = 0.34; Fig. 7). Pigs in the 100%-LPS treatment group spent less (*P* < 0.001) time at the feeder than pigs in the 50%-LPS and SAL treatment groups, and pigs in the 50%-LPS treatment group spent less (*P* < 0.001) time at the feeder than pigs in the SAL treatment group. Within this period, all three treatment groups differed (*P* < 0.001) on days 10 and 11. On days 12 and 13, pigs in the 100%-LPS treatment group spent less (*P* < 0.001) time at the feeder than pigs in the 50%-LPS and SAL treatment groups. There was no treatment difference during the recovery period (*P* = 0.82), with no differences (*P* ≥ 0.90) observed among treatment groups on days 14 to 18; however, on day 19, pigs in both LPS treatment groups spent more (*P* ≤ 0.04) time at the feeder than pigs in the SAL treatment group.

During the early post-recovery period (days 20 to 25), pigs in the 100%-LPS, 50%-LPS, and SAL treatment groups spent 13.0, 13.0, and 11.9% of daily time at the feeder, respectively (SEM = 0.35). During this early post-recovery period, pigs in the 100%-LPS and 50%-LPS treatment groups spent more (*P* ≤ 0.04) time at the feeder than pigs in the SAL treatment group, with no difference between the 100%-LPS and 50%-LPS treatment groups (*P* = 1.00). Within this period, pigs in 100%-LPS and 50%-LPS treatment groups spent more (*P* ≤ 0.04) time at the feeder than pigs in the SAL treatment group on days 20, 21, 23, and 24, with no difference (*P* ≥ 0.39) between the 100%-LPS and 50%-LPS treatment groups. There were no differences (*P* ≥ 0.31) among the three treatment groups on days 22 and 25. During the late post-recovery period (days 26 to 42), there was no treatment difference (*P* = 0.57), and no differences (*P* ≥ 0.81) were observed among across the three treatment groups on individual days (Fig. 7).

### Resting behaviors

#### Time lying lateral

**Figure 8:**
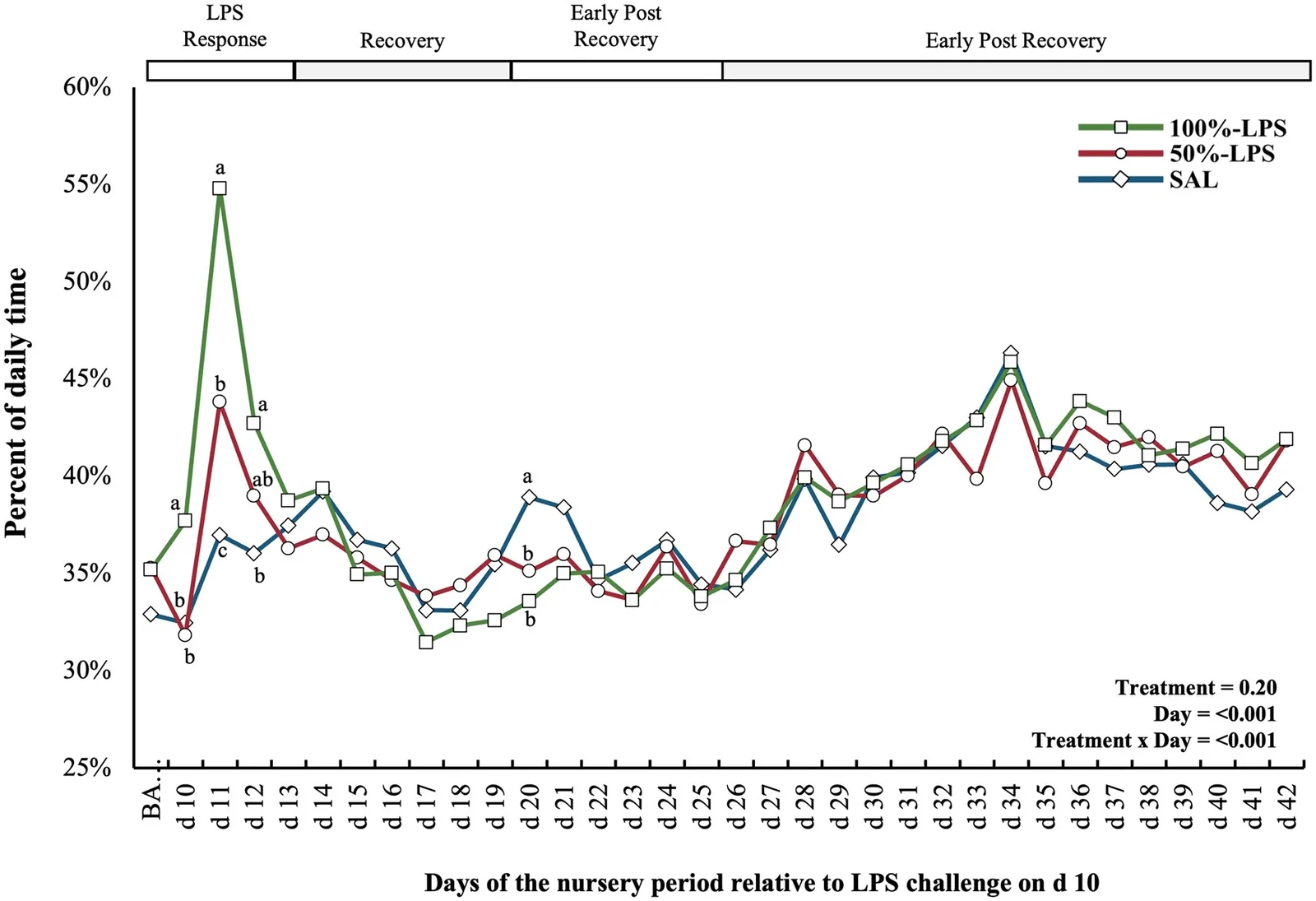
Time spent lying lateral/day by nursery pigs assigned to 100%-LPS, all pigs in the pen received 300 µg/kg BW lipopolysaccharide; 50%-LPS, pens contained a 1:1 ratio of LPS challenged to saline injected pigs; or SAL, all pigs in the pen received saline, across the 42-d nursery period.

There was an effect of day (*P* < 0.001) and a treatment × day interaction (*P* < 0.001) for the percentage of daily time spent lying laterally; however, there was no treatment effect (*P* = 0.20). When evaluated across the four post-challenge periods, there were effects of treatment (*P* = 0.02), period (*P* < 0.001), and a treatment × period interaction (*P* < 0.001). During the baseline period (days 1 to 9), there were no differences (*P* ≥ 0.98) among treatment groups in the percentage of daily time spent lying laterally. During the LPS response (days 10 to 13), pigs in the 100%-LPS, 50%-LPS, and SAL treatment groups spent 44.4, 38.2, and 35.7% of daily time lying laterally, respectively (SEM = 0.56; Fig. 8). Pigs in the 100%-LPS treatment group spent more (*P* < 0.001) time lying laterally than pigs in the 50%-LPS and SAL treatment groups, and pigs in the 50%-LPS treatment group spent more (*P* = 0.004) time lying laterally than pigs in the SAL treatment group. Within this period, pigs in the 100%-LPS treatment group spent more (*P* < 0.001) time lying laterally than pigs in the 50%-LPS and SAL treatment groups on day 10. All three treatment groups differed (*P* < 0.001) on day 11, with the 100%-LPS treatment group spending the greatest percentage of time lying laterally, followed by the 50%-LPS and SAL treatment groups. On day 12, pigs in the 100%-LPS treatment group spent more (*P* < 0.001) time lying laterally than pigs in the SAL treatment group, whereas pigs in the 50%-LPS treatment group were intermediate. There were no differences (*P* ≥ 0.20) amoung three treatment groups on day 13. During the recovery period, there was no treatment difference (*P* = 0.30), and no differences (*P* ≥ 0.53) were observed among treatment groups on individual days 14 to 19.

During the early post-recovery period (days 20 to 25), pigs in the 100%-LPS, 50%-LPS, and SAL treatment groups spent 34.5, 34.8, and 37.2% of daily time lying laterally, respectively (SEM = 0.58). Pigs in the 100%-LPS and 50%-LPS treatment groups spent less (*P* = 0.008) time lying laterally than pigs in the SAL treatment group, whereas the two LPS treatment groups did not differ (*P* = 0.92). Within this period, pigs in both LPS treatment groups spent less (*P* ≤ 0.003) time lying laterally than pigs in the SAL treatment group on day 20, with no differences (*P* ≥ 0.41) observed among treatment groups on days 21 to 25. During the late post-recovery period (days 26 to 42), there was no treatment difference (*P* = 0.57), and no differences (*P* ≥ 0.28) were observed among treatment groups on individual days (Fig. 8).

#### Time lying sternal

**Figure 9:**
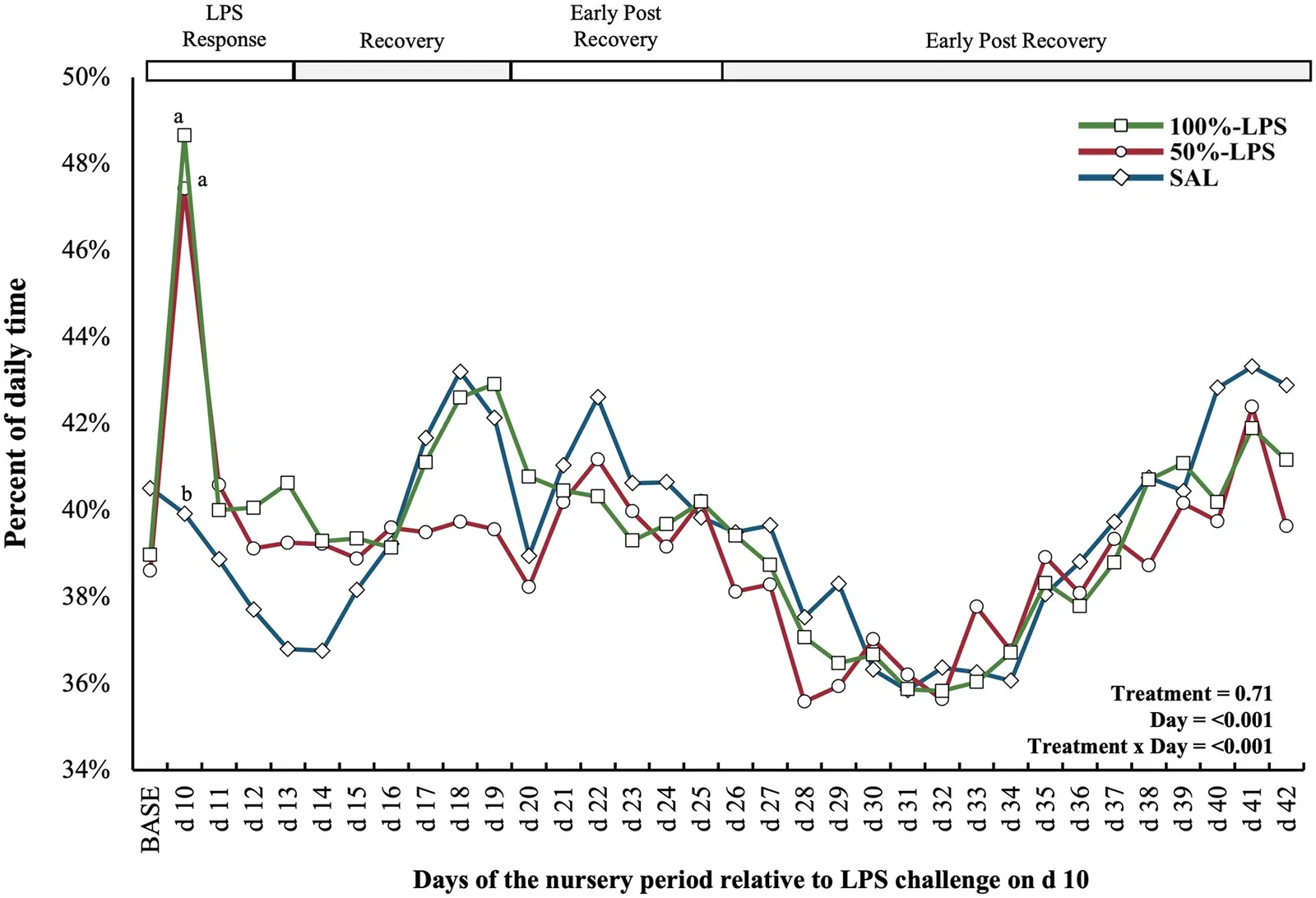
Time spent lying sternal/day by nursery pigs assigned to 100%-LPS, all pigs in the pen received 300 µg/kg BW lipopolysaccharide; 50%-LPS, pens contained a 1:1 ratio of LPS challenged to saline injected pigs; or SAL, all pigs in the pen received saline, across the 42-d nursery period.

There was an effect of day (*P* < 0.001) and a treatment × day interaction (*P* < 0.001) for the percentage of daily time spent lying sternal; however, there was no treatment effect (*P* = 0.71). When evaluated across the four post-challenge periods, there was an effect of period (*P* < 0.001) and a treatment × period interaction (*P* < 0.001), but no treatment effect (*P* = 0.69). During the baseline period (days 1 to 9), there were no differences (*P* = 0.16) among treatment groups. During the LPS response period (days 10 to 13), pigs in the 100%-LPS, 50%-LPS, and SAL treatment groups spent 41.6, 41.7, and 38.6% of daily time lying sternal, respectively (SEM = 0.51; Fig. 9). Pigs in both LPS treatment groups spent more (*P* < 0.001) time lying sternal than pigs in the SAL treatment group, whereas the two LPS treatment groups did not differ (*P* = 0.99). Within this period, pigs in both LPS treatment groups spent more (*P* < 0.001) time lying sternal than pigs in the SAL treatment group on day 10, whereas there were no differences (*P* ≥ 0.07) among treatment groups on days 11 to 13. There was no treatment difference during the recovery period (*P* = 0.33); however, pigs in the SAL treatment group spent more (*P* = 0.005) time lying sternal than pigs in the 50%-LPS treatment group on day 18. On day 19, pigs in the 50%-LPS treatment group spent less (*P* ≤ 0.04) time lying sternal than pigs in both the 100%-LPS and SAL treatment groups. There was no treatment difference during the early post-recovery period (*P* = 0.86), and no differences (*P* ≥ 0.08) were observed among treatment groups on individual days 20 to 25. During the late post-recovery period, there was no treatment difference (*P* = 0.39); however, pigs in the SAL treatment group spent more time lying sternal than pigs in both LPS treatment groups on day 40 (*P* ≤ 0.02) and more (*P* = 0.008) time lying sternal than pigs in the 50%-LPS treatment group on day 42. No differences were observed among treatment groups on the remaining days of the late post-recovery period (*P* ≥ 0.07; Fig. 9).

#### Total time lying

**Figure 10:**
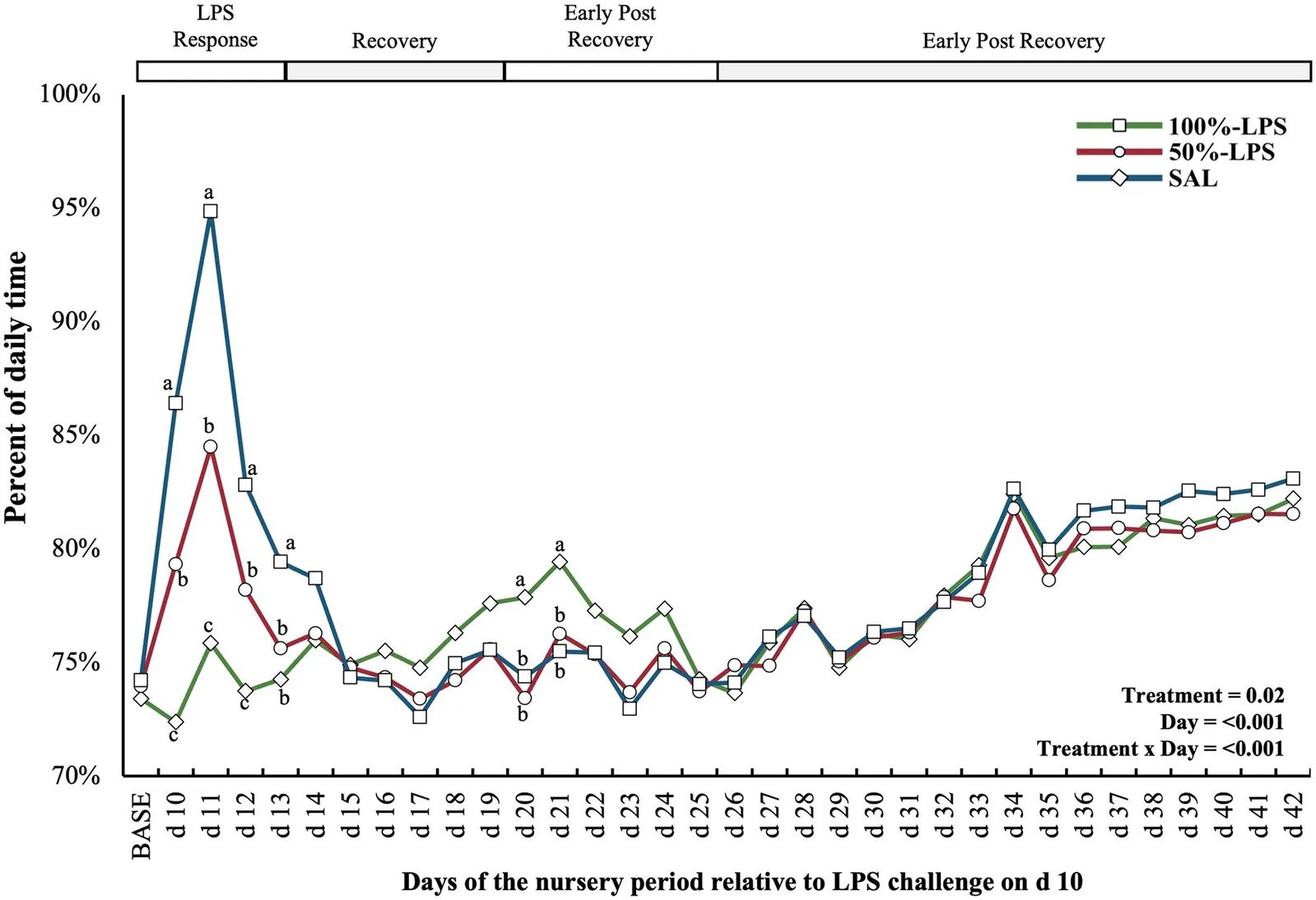
Total time lying/day by nursery pigs assigned to 100%-LPS, all pigs in the pen received 300 µg/kg BW lipopolysaccharide; 50%-LPS, pens contained a 1:1 ratio of LPS challenged to saline injected pigs; or SAL, all pigs in the pen received saline, across the 42-d nursery period.

There were effects of treatment (*P* = 0.02), day (*P* < 0.001), and a treatment × day interaction (*P* < 0.001) for the total percentage of daily time spent lying, calculated as the sum of lateral and sternal lying. When evaluated across the four post-challenge periods, there were effects of treatment (*P* < 0.001), period (*P* < 0.001), and a treatment × period interaction (*P* < 0.001). During the baseline period (days 1 to 9), there were no differences (*P* > 0.99) among treatment groups. During the LPS response (days 10 to 13), pigs in the 100%-LPS, 50%-LPS, and SAL treatment groups spent 86.0, 79.9, and 74.2% of daily time lying, respectively (SEM = 0.43; Fig. 10). Pigs in the 100%-LPS treatment group spent more (*P* < 0.001) time lying than pigs in the 50%-LPS and SAL treatment groups, and pigs in the 50%-LPS treatment group spent more (*P* < 0.001) time lying than pigs in the SAL treatment group. Within this period, pigs in both LPS treatment groups spent more (*P* < 0.001) time lying than pigs in the SAL treatment group on days 10 to 12. On day 13, pigs in the 100%-LPS treatment group spent more (*P* < 0.001) time lying than pigs in the 50%-LPS and SAL treatment groups. There was no treatment difference during recovery (*P* = 0.09), and no differences (*P* ≥ 0.48) were observed among treatment groups on individual days 14 to 19.

During the early post-recovery period (days 20 to 25), pigs in the 100%-LPS, 50%-LPS, and SAL treatment groups spent 74.4, 74.7, and 77.4% of daily time lying, respectively (SEM = 0.44). Pigs in both LPS treatment groups spent less (*P* < 0.001) time lying than pigs in the SAL treatment group, whereas the two LPS treatment groups did not differ (*P* = 0.87). Within this period, pigs in both LPS treatment groups spent less (*P* ≤ 0.03) time lying than pigs in the SAL treatment group on days 20 and 21. There were no differences (*P* ≥ 0.89) among treatment groups on day 22. On day 23, pigs in the 100%-LPS treatment group spent less (*P* ≤ 0.04) time lying than pigs in the SAL treatment group, whereas pigs in the 50%-LPS treatment group were intermediate. There were no differences (*P* ≥ 0.54) among treatment groups on days 24 and 25. During the late post-recovery period (days 26 to 42), there was no treatment difference (*P* = 0.63), and no differences (*P* ≥ 0.54) were observed among treatment groups on individual days (Fig. 10).

#### Time sitting

There was an effect of day (*P* < 0.001) and a treatment × day interaction (*P* < 0.001) for the percentage of daily time spent sitting; however, there was no treatment effect (*P* = 0.49). When observations were summarized by post-challenge period, there were effects of treatment (*P* = 0.04) and period (*P* < 0.001); however, there was no treatment × period interaction (*P* = 0.08). Across the four post-challenge periods, pigs in the 100%-LPS, 50%-LPS, and SAL treatment groups spent 1.2, 1.5, and 1.5% of daily time sitting, respectively (SEM = 0.12).

**Figure 11:**
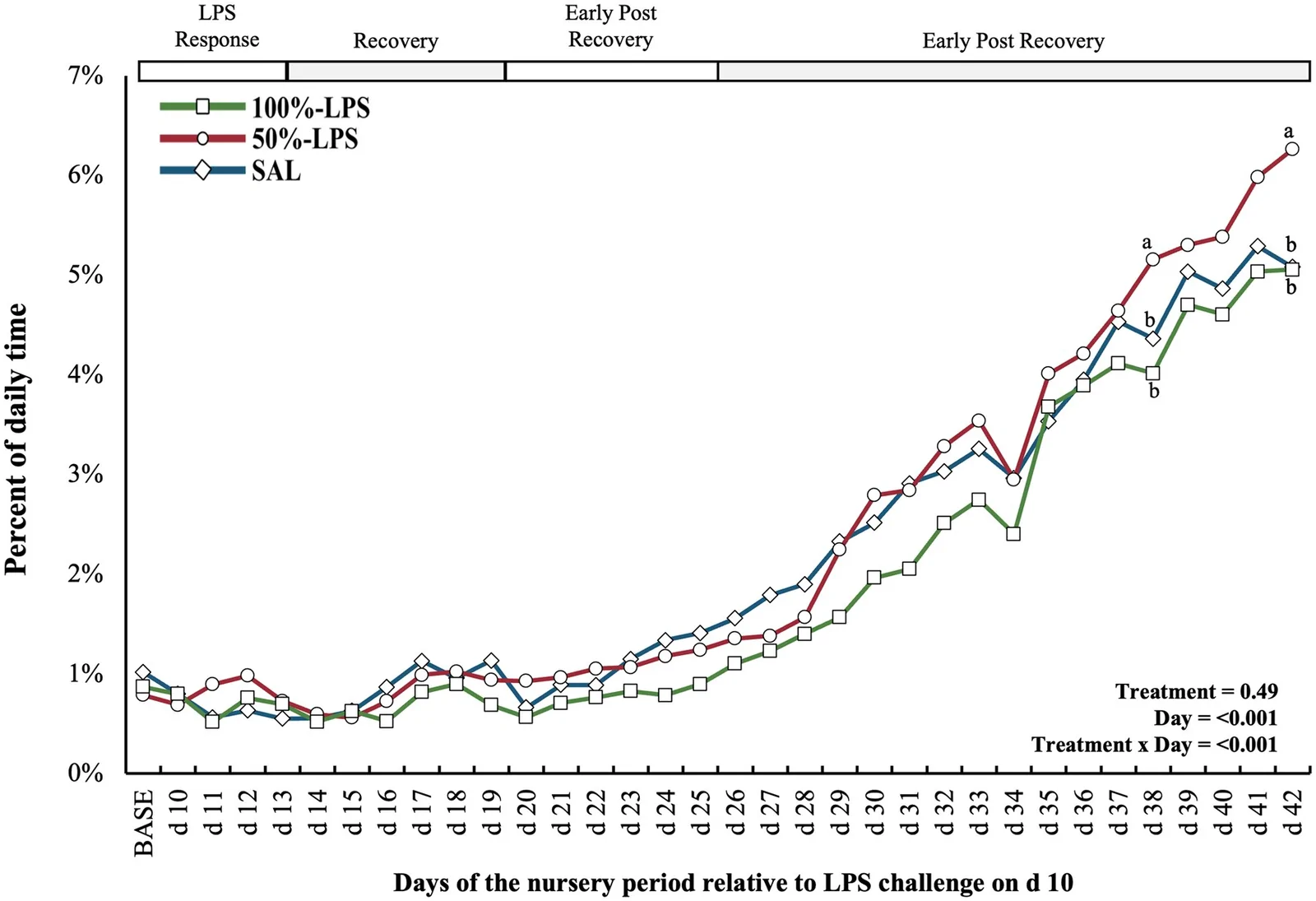
Time spent sitting/day by nursery pigs assigned to 100%-LPS, all pigs in the pen received 300 µg/kg BW lipopolysaccharide; 50%-LPS, pens contained a 1:1 ratio of LPS challenged to saline injected pigs; or SAL, all pigs in the pen received saline, across the 42-d nursery period.

During the baseline period, there were no differences (*P* = 0.83) among treatment groups. The percentage of daily time spent sitting increased across the nursery period, accounting for less than 1% of daily time through day 22 and increasing to approximately 4% by day 35 and 5.5% by day 42. There were no differences among treatment groups on individual days 10 to 37 or days 39 to 41. On day 38, pigs in the 50%-LPS treatment group spent more (*P* = 0.014) time sitting than pigs in the 100%-LPS treatment group, whereas pigs in the SAL treatment group were intermediate. On day 42, pigs in the 50%-LPS treatment group spent more (*P* ≤ 0.012) time sitting than pigs in the 100%-LPS and SAL treatment groups (6.3, 5.1, and 5.1% of daily time, respectively; Fig. 11).

### Performance

There was no treatment effect on weaning BW (*P* = 0.76), with pigs in the SAL, 50%-LPS, and 100%-LPS treatment groups weighing 5.8, 5.7, and 5.7 kg, respectively (SEM = 0.25; Table 2). Nursery exit BW differed among treatment groups (*P* = 0.04). Pigs in the 100%-LPS treatment group had lower (*P* ≤ 0.05) nursery exit BW than pigs in the SAL treatment group (24.3 vs. 25.9 kg), whereas pigs in the 50%-LPS treatment group were intermediate (25.6 kg) and did not differ from either treatment group.

**Table 2:** Nursery performance data of newly weaned nursery pigs exposed to an LPS challenge or sham injection.

	SAL	50%-LPS	100%-LPS	SEM	*P*-value
**Weaning (kg)**	5.8	5.7	5.7	0.25	0.76
**Nursery Exit (kg)**	25.9^a^	25.6^ab^	24.3^b^	0.70	0.04
**Weight Gain (kg)**	20.1^a^	20.0^a^	18.6^b^	0.47	0.02
**ADG (kg)**	0.47^a^	0.47^a^	0.43^b^	0.01	0.01

a,b Within a row, LSMeans without common superscript differ (*P* ≤ 0.05).

Overall weight gain also differed among treatment groups (*P* = 0.02). Pigs in the 100%-LPS treatment group gained less (*P* ≤ 0.05) weight than pigs in the SAL and 50%-LPS treatment groups (18.6 vs. 20.1 and 20.0 kg, respectively), whereas the SAL and 50%-LPS treatment groups did not differ. Similarly, ADG differed among treatment groups (*P* = 0.01). Pigs in the 100%-LPS treatment group had lower (*P* ≤ 0.05) ADG than pigs in the SAL and 50%-LPS treatment groups (0.43 vs. 0.47 and 0.47 kg/d, respectively), whereas the SAL and 50%-LPS treatment groups did not differ.

## Discussion

Sickness behavior is characterized by alterations in posture, behavior, and activity and is one of the most sensitive early visual indicators of illness (Matthews et al. 2016; Bortoluzzi et al. 2023). These behaviors, including lethargy, reduced exploration, decreased feed intake, and increased recumbency, are driven in part by the systemic release of proinflammatory cytokines during immune activation (Dantzer 2001; Konsman et al. 2002; Dantzer et al. 2008). Proinflammatory cytokines along with other inflammatory mediators such as prostaglandin *E*_2_, act on both peripheral tissues and the central nervous system to alter appetite, posture, and voluntary activity (O’Banion 2010; Saper et al. 2012; Skelly et al. 2013). Collectively, these and other immune mediators promote behavioral changes that help conserve energy and redirect resources toward the immune response, providing a central mechanistic explanation for altered pig posture, behavior, and activity during acute disease events.

The LPS model was selected for the present study because it provides a controlled means of inducing short term systemic immune activation and the associated behavioral changes in pigs. This model is well established in swine and is known to induce a robust and predictable inflammatory response. Williams et al. (2009) and Touchette et al. (2002) both reported that TNF α, IL 1β, and IL 6 concentrations increase rapidly following LPS injection, peaking within 1 to 3 h and remaining elevated for several hours after the challenge. In addition to these cytokines, LPS challenge in pigs has also been associated with increases in downstream inflammatory mediators such as prostaglandin *E*_2_, which has been reported to contribute to the broader acute phase response and related behavioral and physiological changes (Liu 2003; Wyns et al. 2015). These inflammatory dynamics are consistent with the behavioral patterns observed in the current study and support the use of LPS as a model of temporary immune activation in pigs. However, cytokines, body temperature, acute-phase proteins, and other physiological indicators of inflammation were not measured; therefore, the magnitude and duration of the immune response could not be directly related to the observed behavioral changes. Collectively, previous studies demonstrate that immune-mediated effects on behavior are measurable and repeatable, supporting the use of the LPS model for evaluating health-related changes in livestock behavior.

Consistent with this inflammatory cascade, the present study documented immediate and pronounced alterations in both active and resting behaviors in pigs exposed to the LPS treatments. During the first 3 d following the LPS challenge (days 10 to 12), pigs in both the 100%-LPS and 50%-LPS treatment groups exhibited substantial reductions in activity, with selected behavioral differences persisting through day 13. Specifically, pigs in the two LPS treatment groups walked fewer meters, completed fewer body revolutions, and spent less time standing and at the feeder than pigs in the SAL treatment group. At the same time, resting behaviors, including time spent lying laterally, lying sternal, and total time lying, increased in the LPS treatment groups, indicating that the challenge shifted behavior away from locomotion and feeder-related activity and toward rest.

These results agree with earlier studies reporting that immune challenge can reduce activity and feeding related behaviors in pigs. Veit et al. (2020) reported reduced activity following LPS challenge, and Escobar et al. (2007) similarly reported reduced locomotor activity and feed directed behaviors in pigs with respiratory disease. However, the present study builds upon those findings by providing continuous behavioral data throughout the entire nursery period rather than relying on isolated time points or limited post challenge observation windows. In the study by Veit et al. (2020), activity was evaluated using scan sampling every 5 min for 6 h after challenge, whereas Escobar et al. (2007) evaluated behavior at 15 min intervals during 3 h observation periods. In contrast, the NU*track* system used in the present study continuously monitored pigs (24 h/d) across the 42-d nursery period, providing an objective assessment of how LPS challenge altered behavior and allowing for evaluation of both the immediate effects of challenge and the dynamic changes that emerged over time.

The behavioral responses observed in the 50%-LPS treatment group also warrant consideration. The 50%-LPS treatment represented a mixed-pen environment in which challenged and saline-injected pigs remained housed together. Behavioral outcomes were analyzed at the treatment-group level, and challenged and saline-injected pigs within these pens were not analyzed separately. With only four 50%-LPS pens, the present study was not designed to independently estimate the effect of LPS challenge status within the mixed-treatment pens. Consequently, the behavioral patterns observed in the 50%-LPS treatment may reflect both the direct effects of LPS challenge in treated pigs and indirect effects associated with sharing the same physical and social environment. The present study cannot distinguish the relative contributions of these effects. This treatment structure may nevertheless better reflect the complexity of group housing, where illness is rarely distributed uniformly among all pigs within a pen.

The present study was designed to characterize continuous behavioral responses to an LPS challenge rather than reconfirm the physiological response to LPS, which has been well established in pigs. Previous studies have demonstrated that LPS challenge induces rapid increases in proinflammatory cytokines and other indicators of acute immune activation (Touchette et al. 2002; Williams et al. 2009). In contrast, less is known about how group-housed pigs alter active and resting behaviors across an extended post-challenge period. To preserve the integrity of these behavioral measurements, cytokines, acute-phase proteins, and body temperature were not measured because repeated blood collection, restraint, or rectal temperature measurement would have disrupted the continuous behavioral responses being quantified. Therefore, the observed behavioral patterns should be interpreted as behavioral responses consistent with the established porcine response to LPS challenge, rather than as direct measures of specific inflammatory mediators or physiological changes.

Although the behavioral patterns observed in the present study are consistent with an acute sickness response followed by later changes in behavior, caution is warranted when interpreting the mechanism responsible for this shift. The early behavioral response aligns with the established temporal pattern of LPS-induced inflammatory activation in pigs (Touchette et al. 2002; Williams et al. 2009), with pigs in the LPS treatment groups initially reducing active behaviors and increasing resting behaviors during the LPS response period. After this acute response subsided, pigs in the LPS treatment groups exhibited a later increase in active behaviors relative to pigs in the SAL treatment group. This later increase was not isolated to a single behavioral outcome but occurred across multiple active behaviors (meters walked, body revolutions, time standing, and time at the feeder) during the same post-acute window (days 19–25), indicating that the LPS challenge was followed by a coordinated shift in active behaviors. Although this later response indicates that pigs in the LPS treatment groups became more active after the acute sickness response subsided, the reason for this change cannot be determined from the present study. The increase may reflect continued recovery from the LPS challenge, greater time spent at the feeder after the earlier reduction in activity, changes in behavior among penmates, or a combination of these or other factors. Because individual feed intake was not measured, increased time at the feeder should not be interpreted as direct evidence of increased feed intake. Despite these later increases in activity and time spent at the feeder, pigs in the 100%-LPS treatment group had reduced ADG, reduced nursery exit BW, and less overall weight gain than pigs in the SAL treatment group, an indication that these later behavioral changes were not accompanied by complete recovery of nursery performance. Future studies that combine continuous behavioral monitoring with measures of feed intake, growth, and physiological status may help clarify the biological basis for these later post-challenge behavioral changes and further define patterns that would likely be missed using shorter and less continuous observation approaches (Escobar et al. 2007; Veit et al. 2020; Bortoluzzi et al. 2023).

In summary, an acute LPS challenge in nursery pigs resulted in clear reductions in active behaviors and increases in resting behaviors during the first 3 to 4 d after challenge, consistent with sickness behavior associated with inflammatory activation. Continuous monitoring also revealed later increases in active behaviors in the LPS treatment groups, including increased walking, body revolutions, standing time, and time spent at the feeder. These later changes were temporally distinct from the acute sickness response and were detected across multiple measures of activity and posture. However, because feed intake, physiological recovery, and the mechanisms underlying these behavioral changes were not measured, it cannot be determined whether the later response reflected recovery, post-challenge behavioral adjustment, altered pen dynamics, or a combination of these factors. Together, these results demonstrate the value of continuous behavioral monitoring for characterizing temporal behavioral changes following immune challenge in nursery pigs and provide a basis for future studies designed to determine the biological mechanisms underlying later post-challenge behavioral responses.

## List of abbreviations

AR(1): Autoregressive 1
IACUC: Institutional Animal Care and Use Committee
IL: Interleukin
LPS: Lipopolysaccharide
NU*track*: NU*track* livestock monitoring system
PRRS: Porcine Reproductive and Respiratory Syndrome
TNF: Tumor Necrosis Factor
